# Can responsible leaders transmute sustainability & OCBE among manufacturers in developing economy? A mediated moderated approach for organizational sustainability

**DOI:** 10.1186/s40359-024-02235-1

**Published:** 2025-01-27

**Authors:** Guohua Wang, Qurat ul Ain Aslam, Naveed Mushtaq, Ayesha Liaqat, Fahad Asmi

**Affiliations:** 1 Zhejiang Conservatory of Music, Hangzhou, Zhejiang People’s Republic of China; 2 Department of Commerce, University of Minawali, Minawali, Pakistan; 3 Malik Firoz Khan Noon Business School, University of Sargodha, Sargodha, Pakistan; 4 School of Business, Charles Sturt University, Bathurst, NSW Australia

**Keywords:** Responsible leadership, Sustainability, Organization citizenship, Employee behaviour, Co Worker Exchange

## Abstract

This study examined the link among environmentally conscious organizational citizenship behavior (OCBE) and responsible leadership among 167 management-level workers in manufacturing plants of arts industry in a developing economy. The study explored the impact of responsible leadership on OCBE, both directly and indirectly through colleague exchange. It also explored the role of a green organizational environment, employee goal orientation, and supervisory support in regulating the link between coworker exchange and OCBE. The findings indicated that responsible leadership held a substantial and favorable influence on organizational citizenship behavior and that this link was mediated via colleague interchange. Workers who exhibited high degrees of goal orientation and were exposed to a sustainable work environment exhibited higher organizational citizenship behavior towards the environment (OCBE), suggesting a connection between colleague contact and OCBE. However, the support from supervisors did not have any moderating effect on this association. The study offers practical and management insights into how to encourage eco-behavior in the workplace.

## Introduction

 The swift industrialization of the global economy has been seen as an important driver of climate change [89, 90, 97]. Companies in these industrialized economies primarily focus on exploiting natural energy resources, altering natural systems from their pristine states, and causing overall environmental deterioration and rapid resource depletion [97]. Local and large-scale construction projects have led to an overload in production, making this industry less amenable to sustainability due to its high consumption of gas and coal [6, 59, 100, 101]. Presently, it is one of the leading industrial producers of carbon dioxide (CO_2_) and is responsible for 5% of all greenhouse gas emissions [6, 17, 72, 101]. The International Energy Agency Report 2023 states, production is projected to increase from 4160 Milestones in 2020 to 4260 Milestones in 2050, with CO_2_ (Carbon Dioxide) emissions expected to rise to 1395 Mt in 2050, positioning this industry as a potential global threat to the environment and a subject of heated debate, especially as world economies transition via the SDGs to the Millennium Development Goals [47]. Organizations grappling with these environmental challenges are hastily adopting environmental policies and practices to enhance their environmental performance [9], pressured by the public and ecological groups to prioritize environmental protection policies [18, 28, 111, 160]. Some scholars working in the same research domain have challenged past scholars’ assertions, arguing that highly rigid environmental rules and regulations are insufficient, and instead, employees’ positive response to environmental concerns is crucial for improving environmental performance [68, 75, 148]. Hence, there is a need for environmental practices among employees, such as Organizational Citizenship Behavior for the Environment (OCBE), which entails free will initiatives with a focus on greener ecosystems [84, 42, 73, 162]. OCBE helps organizations in achieving sustainable development goals, however, such behaviors are relatively rare. Second, the issue has intensified in recent years as practitioners have become increasingly concerned about how to encourage these pro-environmental behaviors in employees [1]. Previous literature [82] has highlighted leadership as a key determinant of employee environmental behavior [3, 94], as it is inherently influential [158]. Prior research has focused on the traditional types of leadership including transformational [14, 61, 146], ethical [74, 164], and charismatic [147], whereas the contributions of RL and the absence of this topic are a conspicuous research gap in the current literature [103, 146]. RL is defined as practice of leadership that incorporates responsibility for the consequences into the processes [8] that affect the organization and the external environment [155]. Transformational and charismatic are conventional types of leadership that target follower transformation specifically in organizational setting by targeting leaders and followers for performance and change. In contrast, RL not only focuses on accomplishing organizational objectives but also on introducing sustainable and responsible practices that ensure stable and positive social and ecological value [96].

Ethical leadership is synthesized within social interactions between two people who are interdependent and influence each other (for example [96]), , is defined as a set of actions based on moral values. Whereas ethical leadership fosters ethical workplace behavior but practicing environmental sustainability would not get a nod of appreciation unless it falls under the puritanical sphere of ethics [116]. RL, however, encompasses a values, norms and principles e.g [112]. , that are specifically embraced and provides for stakeholder engagement both internal and external to champion for the advancement of sustainability development calling for issues like establishing trust, ethically decision making and be environmentally proactive [51, 52, 167].

RL therefore helps OCBE by ensuring that accountability to the environment is integrated in the central operations, encouraging conspicuous green behavior within organizations as well as enhancing support of sustainable development objectives. OCBE is best addressed by this approach because the management of environmental responsibility is incorporated into its basic framework, which is different from other traditional styles like transformational and charismatic leadership. Therefore, RL is more beneficial to OCBE as compared to other leadership modes of operation that are targeted at ethical practice or firm change and presents structures that support long-term responsible organizational practice [157].

Therefore, this study considers RL as an important antecedent of OCBE as it emphasizes social responsibility and stakeholder well-being. For sustainable development and business success, it is not enough to focus only on the bottom line. Leaders must also recognize the importance of engaging employees in socially responsible practices [102, 167]. Numerous studies have shown that leadership influences employees’ pro-environmental behavior [51, 165]. So, this study fills this literature gap by exploring the link between RL and OCBE and its mechanism. By delving into the mediating function of coworker exchange, the results of this study expand upon previous understandings of leadership and OCBE. According to [131], there are two primary relationships in the workplace: both the leader-follower dynamic in a social setting and the dynamics between employees who together form a system of cooperation, coordination, and synergy in organizations. RL based on values often involves others in achieving organizational outcomes. Therefore, this study proposes coworker exchange as a mediator between RL and OCBE [129]. define coworker exchange as “a dynamic peer relationship between employees who report to the same manager”.

This paper examines relationships in organizations through the view of Social Exchange Theory (SET) as defining relationships as a process of reciprocal exchange where individuals engage in behaviors that will benefit both parties [21]. In the context of SET it is proposed that people process interactions in terms of potential rewards and costs with the goal of achieving the maximal balance. Interactions that are characterized as positive; that is, those that include trust, respect, and support produce feelings of obligation, which translates to improved levels of cooperation, participation, and organizational commitment [64]. Interestingly, SET has been useful in accounting for OCBs, demonstrating how trust-based relations prompt workers to perform more than is expected of them. Furthermore, SET posits that leaders who embrace the right business ethics and have concern for stake holders’ wellbeing create a feeling of responsibility towards the employees and hence should reciprocate in a way that is desirable to organizational and environmental objectives. This makes SET particularly relevant for the purpose of this study, Organizational Citizenship Behavior for the Environment (OCBE), because it is more likely to be experienced by the workforce where the employee is encouraged and recognized by their leaders and other employees.

Moreover, coworker exchange is the mediator that proves that RL has an indirect relationship to OCBE. SET assists this mediation by underscoring that employees who are positively related with their coworkers; are motivated to assist their counterparts, provide them with emotional and informational support, as well as giving constructive criticism making it easier to adapt a culture of togetherness (for instance [25, 88]). Research that has employed SET has found that coworker support means that people get compelled to reduce the stress that is consequent on their workload and this they achieve by handling personal issues for one another and sharing responsibilities, that is, there is a reciprocal exchange of resources that is mutually beneficial within the organization and for the individual e.g [102, 156]. As a result of coworker exchange, subordinate employees emulate the self-responsible behaviors that have been instilled by organizational leaders, thus promoting a culture of togetherness and environmental stewardship in workplace interactions. That is why there is a high level of coworker exchange, creating a network in which mutual support and sharing of resources become commonplace, supporting the connection between responsible leadership and OCBE [108]. In this way, the positive effects of peer dynamics that occur through coworker exchanges indirectly support the implementation of responsible leadership’s pro-environmentalism values in the behavior of the workers. Therefore, coworker exchange is a mechanism through which the effect of responsible leadership on OCBE is cascaded, showing that positive relationships with peers are central to development of RL standards in organizational culture.

This research also emphasizes and builds upon SET to establish a theoretical framework that identifies employee goal orientation, GROC, and the supervisory support as the variables that moderate the relationship between coworker exchange and OCBE [32, 138]. According to SET, people involve themselves in interpersonal relationships with the purpose of receiving mutual benefits as the costs and benefits of each interaction are carefully evaluated [21]. In an organizational context, coworker exchange therefore emerges as a critical process through which RL is associated with OCBE. However, the nature of the coworker exchanges, which lead to the realization of OCBE, is contingent on contextual and individual aspects like goal orientation, GROC and supervisory support.

Several studies have also suggested that OCB may actually serve as a demanding job aspect, as is the case with OCBE, and hence may be likely to consume resources from employees across their formal and informal organizational roles [113, 159]. In such instances, psychological resources from the supervisor level can help to restore/recharge up the employee’s resources and provide encouragement to the employee to perform voluntary actions like OCBE [78].

These behaviors can be summarized as establishing and maintaining a high degree of trust and commitment between the employees and supervisors to ensure they are surrounded by a positive support environment whereby they will want to be of positive utility to the organization on issues concerning the environment [110]. This study therefore seeks to establish how supervisory support moderates the relationship between coworker exchange and OCBE to highlight the impact of managerial encouragement towards environmental practice.

Another important factor is the context factors, for example organizational culture which also facilitates or constrain employee behavior. Environmental psychologists and organizational researchers have also stressed that organizational factors act as constraints into the expression/experience of voluntary actions [42, 43, 87, 125]. In particular, an organization’s green organizational climate, or GROC, proposes the extent to which green policies, processes and practices are valued and endorsed by the staff [113]. OCBE can be reciprocated readily in a supportive green climate with coworkers’ interactions because employees are inclined to think that such behaviors are consistent with the organizational green environment. It is thus possible to conclude that a well-developed GROC enhances beneficial impact of coworker exchange on OCBE [46, 89, 169].

Additionally, goal orientation brings out an individual level factor that influences an employee’s behavior in relation to social exchanges within the workplace. According to earlier research in organizational psychology, goal orientation such as learning, and performance orientations significantly forecast task and non-task work behavior [91]. Workers with high goal orientation should perceive coworker exchange as a positive experience that fosters learning and their motivation to return such experience through OCBE should be high. Interestingly, the moderation of OCBE by goal orientation is an understudied area awaiting more exploration of how goal-oriented employees will be to environment citizenship initiative. This study fills this gap by developing the hypothesis that goal orientation mediates the relationship between coworker exchange and OCBE, providing fresh perspective on how self-posed goals can facilitate pro environmental behavior. In this model of SET, the process of the supportive exchange between coworkers and the role of contextual and personal variables in enhancing the coworker exchange-OCBE bond are presented. With employee goal orientation, GROC and managerial support added to the current paper, this study offers a more enhanced understanding of how social exchanges and organizational context interactively induce environmentally responsible behavior of employees within organizations. Therefore, the objective of this research is to examine the relationship between Responsible Leadership (RL), Coworker Exchange (CWX), and Organizational Citizenship Behavior for the Environment (OCBE). It aims to explore CWX’s mediating role in the RL-OCBE relationship and to investigate the moderating effects of Employee Goal Orientation (EGO), Green Organizational Climate (GROC), and Supervisory Support (SS) on the CWX-OCBE link.

## Literature review

### Responsible Leadership (RL) and OCBE

The awareness level regarding sustainable development management is increasing day by day due to environmental degradation [13]. A significant number of academics who have researched and talked about sustainable management in firms have focused on examining citizenship behavior at the strategic level rather than employee environmental behavior [58]. Employee environmental behavior is equally significant in raising the bar for sustainable management at the organizational level on a daily basis, workers are responsible for putting the company’s plans into action [53].

Within an organization, employees participate in a variety of environmental protection practices; however, neither these practices nor the formal reward system of the organization often rewards them. We refer to these practices as OCBE [91]. OCBE could be defined as employee behavior that goes beyond the official boundaries of organizational activities while performing their duties. In this regard, employees perform tasks that are not formally required by the organization [42]. An enterprise must implement this essential strategy for green development, which also serves as a helpful and practical supplement to employees’ environmentally protective behavior [38]. For example, employees in an organization may save paper, consume energy cautiously without wasting it, assist the organization in safeguarding the environment, and help their colleagues adopt green behavior [75, 139, 168]. To meet the criteria for green policy and strategy of the organization, employees engaged in OCBE put their ideas and intentions related to environmental protection into practice [118]. Research and investigation must be undertaken into the antecedents of employees’ sustainable behavior (e.g., citizenship behavior) because it significantly impacts the environmental performance of organizations [80, 142]. Environmental self-responsibility [65, 89] enterprise environmental issues and attitudes [142], workers’ perception of organizational support [80, 109] and organizational environmental protection measures [89] have been recently studied as important factors that lead to OCBE. On the other hand, when it comes to leadership, several studies have shown that environmentally conscious leadership [114, 115] and ethical leadership [166] greatly contribute to improving employees’ OCBE.

The theory of social learning, as stated by [11], suggests that individuals observe and imitate others, thus guiding their behavior. In RL, leaders primarily focus on the interests of various business stakeholders and exchange opinions and information with employees during communication. This behavior leads responsible leaders to convey all pertinent information to employees through interaction. The subordinates not only closely observe but also emulate their leaders by gradually accepting and internalizing their values. Previous research has shown that RL [91], job performance [20, 84, 85], job satisfaction [154], organizational commitment [3, 44, 142], turnover rates [45], and unethical behavior [149, 155] significantly influence organizational citizenship behavior.

Ethical issues are not the sole concern of RL; they also focus on establishing long-term goals and fostering relationships with stakeholders. Organizational citizenship behavior encompasses ideas conforming to the principles of RL [94]. It reflects an individual’s efforts and ethical beliefs aimed at balancing the association between nature and human society while ensuring managerial stability. Responsible leaders advocate for the development of management measures and codes of conduct concerning environmental protection [137]. Additionally, responsible leadership enhances employees’ extra-role performance, such as organizational citizenship behavior [33, 96]. When making decisions, it aims to take everyone’s needs into account.

Such leadership behavior serves as inspiration for employees, prompting them to imitate it and eagerly seize opportunities to care for and assist others, as well as to take the initiative in performing extra-role behaviors. RL has a role-model effect, thus enhancing employees’ organizational citizenship behavior [155]. Subordinates form a relationship with leadership, where leadership’s environmental behavior and concern for environmental protection serve as examples for employees to follow. The concept of RL aligns with organizational citizenship behavior in that it integrates the concepts of leadership and social responsibility, considering stakeholders’ interests [44, 107] while striving to achieve ecological, social, and economic benefits. In conclusion, this study believes that RL aims to maintain the balance between nature and society and is ethically concerned about its responsibility regarding the environment. As a role model, responsible leadership also serves as a source of encouragement for employees to exhibit organizational citizenship behavior.

- H1: Responsible leadership (RL) is positively associated with OCBE.

### Responsible Leadership (RL) and co-worker exchange

We refer to dyadic relationships between employees of the same rank as co-worker exchanges [31, 129]. It refers to the social exchange relationships that employees have with co-workers of the same status [77]. Individual and contextual factors in the workplace influence co-worker exchange. Individual factors, in particular, include individuals’ similarities and personalities, while contextual factors include proximity to work, shared tasks, slack time, and work-related problems [132]. Previous research has found that leadership creates a communication context and provokes frequent interactions among coworkers, increasing co-worker interdependence, support, and cohesion [14, 133, 156].

Furthermore, the balance theory [66] suggests that when a manager has confidence in two employees, it will lead to mutual trust between those workers. Balance theory posits that a system of triadic relationships involving two individuals and one object (thus, three individuals in total) will eventually reach a state of equilibrium. Another way is to say that if an employee feels the same way about their manager as another does about the leader, then activities of that nature will occur in the workplace, eventually bringing the system to a state of balance. According to [85], those co-workers who perceive the behavior of a positive leader tend to view themselves as similar. Therefore, they build a closer relationship with each other. Leaders, through their role as official performance appraisers, distributors of rewards, and very often mentors of subordinates, have the potential to shape the immediate workgroup environment of the workgroup and strengthen employees’ trust in their co-workers [31, 81]. The leader-member exchange theory posits that employees who receive support from their leaders cultivate positive relationships with their co-workers, thereby enhancing individual task performance [14, 23]. To summarize, based on the above literature review and theoretical background, our proposition asserts that RL significantly influences the establishment and sustenance of employee-member exchange within the workplace.

- H2: RL is positively associated with coworker exchange.

### Co-worker exchange and OCBE

In previous literature, researchers have particularly emphasized the importance of co-workers as crucial social referents for various reasons. Primarily, the relationship between employees and their colleagues has become extremely crucial due to the growing trend toward team-based structures in organizations [23]. Thus, there is a strong likelihood that employees influence their colleagues in the workplace [70, 105]. Second, colleagues help to define the workplace environment [35]. Third, employees draw social comparisons with the input-output ratio of their colleagues, which affects their job satisfaction and performance. According to [102], co-workers are highly relevant referents for social comparisons within organizations, which influence individual evaluation in various ways. For these reasons, it is assumed that co-workers have a substantial effect on employees’ work attitudes and performance [129]. In co-worker relationships, individuals have no formal authority over others; this association is based on common liking, resemblance of attitudes [122], or personal choice and initiative. Hence, co-workers are a crucial resource of instrumental and emotional support for employees [131].

Furthermore, coworkers’ roles in the workplace have received more attention [34]. Just a few of studies have looked at the effects of co-worker exchange (CWX) on employee attitudes and behaviors [67, 141]. Research grounded on social learning theory and social exchange theory shows that employees are more inclined to provide emotional support, constructive criticism, knowledge sharing, and physical assistance to one another when they have good relationships with their colleagues [86, 127]. These work qualities positively correlate with facilitating others [7] and job performance [79]. Prior studies [31, 34, 43] (have demonstrated that positive connections with coworkers enhance various aspects of job performance, including commitment to the organization [69], accomplishment of tasks, job satisfaction, and organizational citizenship behaviors.

Employee stress, attrition, and dissatisfaction with work are all decreased when there is a high level of coworker interaction [133]. Furthermore, it is suggested by [43, 156] coworkers with strong supporting behavior are generally more inclined to share the workload and assist their colleagues with personal matters. Since resources and support are exchanged in high-quality coworker exchanges, they can be advantageous for both individuals and companies [88].

Coworker advocacy in environmental work significantly enhances employees’ tendency to exhibit pro-environmental behavior in organizations [128]. Social exchange theory, operating on the reciprocity principle, serves as the foundation for the interpretation of CWX. This principle suggests that individuals in high-quality relationships will act in a manner that will favor their partner, with each party bringing different types of resources to the relationship [85]. In co-worker exchange, employees receive information sharing, help, caring, and support from their co-workers, which often extends beyond work-related matters. Furthermore, coworkers are more likely to interact with employees than executives are, which fosters mutually beneficial relations among coworkers and can have a ripple impact on the firm as a whole [34]. The role of interpersonal dynamics in encouraging eco-friendly actions in the workplace has received little attention from researchers [52, 137] some empirical studies have demonstrated that co-worker exchange significantly impacts employees’ organizational citizenship behavior [77, 78, 95, 140]. Consequently, we expect to observe a positive impact of co-worker exchange on organizational citizenship behavior related to the environment and propose that:

- H3: Co-worker Exchange is positively associated with OCBE.

### Mediation of co-worker exchange

 [170] argued that leaders create an atmosphere conducive to problem-driven, open conversation when they treat their people with honesty, respect, and trust. This type of environment encourages followers to cooperate and care for one another. When employees in an organization receive proper treatment from both their leaders and co-workers, they go beyond their formal duties to contribute to the organization [150]. Social exchange theory and social learning theory-based research consistently demonstrate that individuals with strong relationships with their coworkers are more inclined to aid one another and provide knowledge, emotional assistance, and constructive criticism [86, 127, 129]. In exchange relationships characterized by trust, loyalty, and respect [81], employees are more likely to support their co-workers with high CWX. Consequently, they engage in more environmental behaviors [10]. Leaders who enhance exchange relationships among co-workers ultimately stimulate employees’ citizenship behavior [32, 134]. Based on theories and past empirical evidence, we hypothesize that:

- H4: Co-worker Exchange mediates the relationship between RL and OCBE.

### Supervisor support as a moderator

The connections between employees’ immediate superiors and those under them create a nexus., which causes the emergence of different organizational activities [161]. Several researchers have investigated and focused on understanding and improving these workplace relationships. How supervisors treat their subordinates has been the subject of much attention because it affects the relationships between them and has a positive effect on several job-related outcomes, including task performance, commitment, and organizational citizenship behavior [40, 41]. Perceived supervisor support, or PSS, refers to the general viewpoint of subordinates on the extent to which supervisors value their contribution, take into consideration their well-being, and offer useful and enthusiastic assistance [2]. Additionally, supervisory assistance fosters the growth of bonds between employees and supervisors [21, 25]. According to the social exchange theory, if a trustor believes that the trustee is untrustworthy, the trustee will not participate in social exchange practices [21, 40].

In light of Normand’s theory of social exchange, employees feel compelled to act in their superiors’ best interests when they sense support from them [21]. To repay that favor, they exhibit what is known as OCB behavior, which is supportive and advantageous. Supervisory support improves citizenship behavior [84]. Workers who believe their managers are not supporting them as much exhibit poor citizenship [163] [5]. asserted that supervisors play a crucial role in encouraging their subordinates to reciprocate by exhibiting good citizenship. According to [170], managerial support acts as a beacon to inspire employees to exhibit pro-environmental behavior [90]. A leader’s actions have a huge impact on how their subordinates behave. Therefore, analyzing supervisory support as a moderating variable can improve organizational citizenship behavior for the environment, which is one of the goals of the current study. Consequently, the current study proposed the following hypothesis:

- H5: The relationship between co-worker exchange and OCBE is positively moderated by supervisor support.

### The moderating role of employee goal orientation

When it comes to variances in motivation across people, goal orientation is among the most studied [49, 92]. According to perceptual-cognitive frameworks [6] individuals’ perceptions, understandings, and actions in contexts pertaining to accomplishment are characterized. One definition of goal orientation offered by [48] is “an individual propensity towards developing or validating one’s ability in achievement settings.” [24] further divided it into learning and performance goal orientation. Learning goal orientation is the desire to better oneself by picking up new abilities, adapting to novel circumstances, and learning from new experiences. An orientation toward performance goals is the desire to show others that one is competent and to receive positive feedback on one’s performance [136, 153].

The literature in organizational psychology has investigated and analyzed it as a predictor of task and job performance [151]. Environmental citizenship behavior (OCBE) and other non-task work behaviors may be theoretically predicted by goal orientations, according to this research. Much of the recent research on the antecedents of non-task work behaviors has focused on personality factors [22, 83, 104, 107, 124]. According to [166], workers’ work habits are favorably predicted by goal orientations, such as green practices. Performance goal orientation alters OCBs dramatically, according to [19]. People who work for companies that place a premium on performance goals tend to be more concerned with meeting or exceeding normative performance standards [48, 49]. They desire a positive evaluation of their competence [152] which requires them to exert the necessary effort to exhibit behaviors they believe their managers and organization will appreciate. Since both performance and learning goal orientations are centered on attaining desirable outcomes (such as receiving assessments of normative competence and task mastery, respectively) [48, 49], have argued that both orientations should result in similar positive outcomes [19].

- H6: The relationship between co-worker exchange and OCBE is positively moderated by Employee Goal Orientation.

### The moderating role of organizational green climate (GROC)

The ways people act is greatly affected by the workplaces in which they work. A person’s attitude may be shaped by their social surroundings, as stated in the social information processing hypothesis. What this means is that the people we spend the most time with have a significant impact on our values, attitudes, and behaviors [123]. According to GROC [37], which, to reiterate, refers to employees’ shared perceptions of their organization’s commitment to environmental sustainability. In contrast to more rigid organizational settings, employees prefer to work in more open-ended social settings. Workers decipher the signs and symbols in their physical surroundings; this is how they understand and navigate their workplace. An organization’s atmosphere, defined by [126] as the collective perception of workers about organizational practices, processes, and policies, is formed via this collective sense-making. The GROC, which measures the extent to which employees appreciate the environmental responsibility of their employer, is no different.

 [37] suggests combining specific environmental obligations like recycling, chemical control, and water resource management with environmental policy and management orientation, which includes things like G. statements, instructions, staff information distribution, and supervisory actions. Workers pay closer attention to what their immediate bosses say and do [25]. Managers do more than only lay out the laws and regulations of the firm [50]; they also provide an example for their workers to follow, motivate them to achieve personal goals, and encourage them when they face challenges outside of work [169]. Thus, the environment, and the climate component in particular, creates a normative framework that signals to workers the organization’s goals and values, and the attitudes and behaviors that workers should display in response. Therefore, organizational environment helps bring about congruence between employee motivation [47] and effort and the organization’s objectives, projects, and ambitions. Therefore, it is reasonable to expect that workers may be more inclined to take part in pro-environmental actions when they perceive that their firm fosters a green atmosphere. Environmentally friendly practices and policies in the workplace inspire people to act and share what they’ve learned [60]. According to [146], when employees perceive support from their work environment, such as adequate resource provision, manager support, and encouragement for exhibiting pro-environmental behavior [158], it both provokes and positively moderates their environmental behavior within the organization.

 [135] found that creating a green workplace helps workers make the link between their own beliefs and the ethics and citizenship of their company. In addition, research has shown that green climate significantly influences organizational citizenship behavior for the environment (OCBE) [169]. According to [89], the GROC shows how well and appropriately behaviors are done, and the integrated expectations make the whole spectrum of employee actions clear. The relationship between green leadership and environmental citizenship actions taken by workers inside an enterprise may be moderated by the atmosphere in which the business operates. The study’s findings suggested that drawing from theoretical understanding and actual data, we hypothesized that:

- H7: The association between co-worker exchange and OCBE is positively moderated by GROC.

## Research methodology

### Scale development

**Fig. 1:**
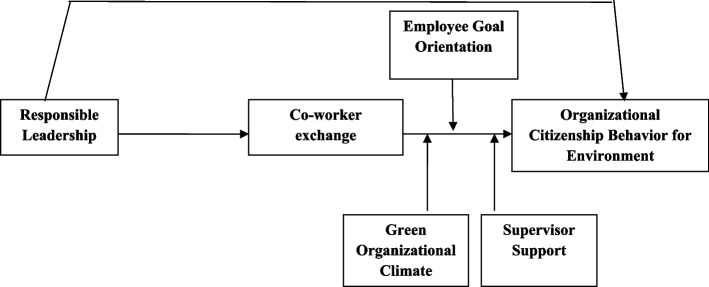
Research framework

We adapted each item of the measuring tool from the available literature so it would work in our investigation. We tailored the scales to the specifics of our study to ensure the analytics managers were using them effectively. Five seasoned academics then verified the validity of the survey’s content. After that, we conducted a survey pilot study with 52 participants from the art department of Sargodha University, Pakistan, with the permission of the Sargodha University Review Board (SURB). However, SURB waived the consent of participants. This made it possible to test the robustness of our suggested model before gathering all the necessary data. We utilized a 5-point Likert scale for all of our items, where 1 signifies strong disagreement and 5 signifies strong agreement (Figure 1).

RL was measured using a scale, consisting of five items developed by [79, 154]. provided the five items used to assess coworker exchange. We used 10 items scale to measure OCBE [167]. Supervisory support has adopted the questionnaire of [78, 143, 144], which contains 16 items. Green climate: Psychological green climate was measured using five items from [46, 110]. To measure the goal orientation of employees, 12 items were used, six-item learning goal orientation scale and the six-item performance goal orientation adopted from scale adapted by [7, 130].

### Data collection

This is a cross-sectional study. In a cross-sectional study you collect data from a population at a specific point in time. The final data was collected from Pakistan’s management-level employees in the cement manufacturing industry. There are currently 17 different manufacturing plants in Pakistan. Carbon emissions from cement industries of Pakistan has increased 17% from 2015 to 2020 [99, 119]. If the emissions rate stays the same, global warming will rise significantly and directly affect the environment drastically. Therefore, there is an important need to practice pro-environmental behaviors to save the ecological system [27]. Different chambers of commerce and industry were contacted for distribution and receiving of the questionnaires back from the respondents. Additionally, for broader purposes of generalization, a true representative sample in the probability sampling design is crucial. Based on the data from the Chambers of Commerce and Industry there were approximately1000-1100 employees working at different managerial levels in the industry design field. Consequently, the sample size was easily ascertained using Krejcie and Morgan’s table, which indicates that a sample size of 370 is needed. A crucial first step was estimating the anticipated response rate. The low response rate in a few prior research studies has been noted. It is also recommended increasing the sample size by 40% to address the risk of sample attrition. So, using simple random sampling, 518 questionnaires were randomly given to managerial-level staff members. A 42.27% response rate was obtained from the 219 surveys that were returned out of the 518 that were sent out. With much effort and commitment on the part of the researcher, the data was collected in nearly four months’ time. A merely 167 completed surveys were deemed useful for additional analysis of the 219 returned surveys; this resulted in a usable response rate of 32.23%. It is pertinent to mention here that we treated each cement manufacturing plant as a separate “stratum.” This ensured that each plant is equally represented in our sample. Since we sent out 518 questionnaires, we divided this number equally as well.

This means sending out around 30 questionnaires per plant (518 / 17 ≈ 30). This accounts for potential non-responses. Within each plant, we selected 30 employees randomly to receive the questionnaire. This was done using simple random sampling within each stratum.

Use of stratified random sampling ensured that each plant is represented equally in the final sample making our findings more generalizable. Out of the completed 167 surveys, descriptives show that 32.2% respondents are 50 years of age or older, 34.8% are between 34 and 41 years of age, and 18.6% and 14.4% of respondents are between 42 and 49 years of age and between 26 and 33 years of age, respectively. Men make up 82.4% of the respondents, compared to women’s 17.6%. According to the data analysis, 32% of respondents have a postgraduate degree (master’s or PhD), 40.4% have an undergraduate degree, and 27-point 6% have a college qualification. 33.6% of respondents indicated that they had worked for their company for two to five years when asked how long they had been there.

### Analysis and findings

According to [146], the research estimates the model using PLS path modeling. Because PLS can provide and evaluate theoretical model explanations and predictions, this work provides justification for its usage. Using PLS, you may sidestep the challenges of constructing a model and determining which components to utilize, as well as dealing with measurement levels, sample sizes, and multivariate normality assumptions [36]. Based on the research conducted by [63], it was shown to be a more suitable technique for hierarchical model forecasting than CBSEM. Following the methods outlined by [120, 121], the model was calculated using Smart PLS 3.0. According to [62], the research calculated standard errors of estimates using a non-parametric bootstrap method with 5000 replications. After calculating first- and second-order component scores with the same number of indicators, we repeated the process in accordance with the principles of hierarchical modeling [16]. A structural equation model (SEM) evaluation consists of two sequential steps: We begin with a thorough analysis of the measurement model and then go on to the structural model.

## Assessment of measurement & structural model

### Measurement model

Based on the criteria laid forth by [35], we ran two sets of tests to make sure the measurement model is convergently valid. As shown in Table 1, where the loading for all items was more than 0.70 at *p* < 0.001, the use of Confirmatory Factor Analysis (CFA) provided evidence for the convergent validity. Following the advice of [34, 54], the second stage in validating the measuring scale was to find the composite reliability (CR) and average variance extracted (AVE). According to Table 1 [62], the CR value is 0.80 and the AVE value is 0.50, which are both over the minimum acceptable level. Every item has a higher loading on its own construct than on any other construct, meaning that there are a lot of variances between each construct and its pieces. This suggests that discriminant validity is supported by cross-loadings. If the values of the square root of the AVE surpass the intercorrelations of the construct with the other constructs in the first-order model, as specified by [54] then the findings are shown in Table 2 and also confirmed through HTMT in Table 3. We computed the variance inflation factor (VIF) to definitively disprove the existence of multicollinearity. Collinearity could not have occurred since the values, which ranged from 1.486 to 2.732, were far lower than the minimum acceptable value of 5.

**Table 1:** Reliability and validity of the instrument

	Loadings (λ)	CA	CR	AVE
CWX1	0.808	**0.861**	**0.90**	**0.644**
CWX2	0.831			
CWX3	0.817			
CWX4	0.824			
CWX5	0.728			
**(Higher Order Construct) EGO**		**0.92**	**0.933**	**0.583**
EGO_LGO1	0.732	**0.877**	**0.907**	**0.620**
EGO_LGO2	0.790			
EGO_LGO3	0.800			
EGO_LGO4	0.779			
EGO_LGO5	0.805			
EGO_LGO6	0.814			
EGO_PGO1	0.872	**0.92**	**0.933**	**0.583**
EGO_PGO2	0.866			
EGO_PGO3	0.792			
EGO_PGO4	0.812			
GROC1	0.818	**0.873**	**0.908**	**0.664**
GROC2	0.817			
GROC3	0.837			
GROC4	0.832			
GROC5	0.769			
LS10	0.774	**0.807**	**0.873**	**0.633**
LS5	0.797			
LS8	0.831			
LS9	0.780			
OCBE10	0.761	**0.862**	**0.897**	**0.593**
OCBE2	0.758			
OCBE4	0.835			
OCBE5	0.816			
OCBE7	0.740			
RL1	0.819	**0.841**	**0.887**	**0.612**
RL2	0.808			
RL3	0.776			
RL4	0.762			
RL5	0.743			

*CA* Cronbach’s Alpha, *CR* Composite Reliability, *AVE* Average Variance Extracted

**Table 2:** Constructs’ discriminant validity (Fornell and Larcker criterion)

	Co-worker Exchange	Green Organization Climate	Learning Goal Orientation	OCTOBER	Performance Goal Orientation	Responsible Leadership	Supervisory Support
Co-worker Exchange	0.802						
Green Organization Climate	0.627	0.815					
Learning Goal Orientation	0.535	0.691	0.787				
OCBE	0.594	0.743	0.697	0.77			
Performance Goal Orientation	0.602	0.65	0.788	0.63	0.836		
Responsible Leadership	0.512	0.571	0.541	0.641	0.524	0.782	
Supervisory Support	0.461	0.535	0.469	0.611	0.468	0.678	0.796

**Table 3:** Constructs’ discriminant validity (HTMT criterion)

	Co-worker Exchange	Green Organization Climate	Learning Goal Orientation	OCBE	Performance Goal Orientation	Responsible Leadership	Supervisory Support	Co-worker Exchange
Co-worker Exchange								
Green Organization Climate	0.66							
Learning Goal Orientation	0.717	0.794						
OCBE	0.605	0.803	0.789					
Performance Goal Orientation	0.679	0.793	0.812	0.8				
Responsible Leadership	0.698	0.832	0.751	0.851	0.733			
Supervisory Support	0.597	0.64	0.665	0.629	0.752	0.616		
Co-worker Exchange	0.548	0.575	0.638	0.556	0.733	0.564	0.824	

CMB is always an important issue in any survey research design and thus would always be of concern in this study [56]. To further avoid having CMB be a problem in our research we guaranteed the respondents anonymity and confidentiality so that he or she would not feel compelled to respond in a way they deemed socially desirable. It is normal for respondents to provide realistic and genuine answers when they know that their answers will not be attributed to them in anyway. Secondly, we randomized the order of questions belonging to different constructs in your survey to minimize the problem of consistency motives. Thirdly, clarify research purpose and instructions, making it clear what the item is for by stating the research purpose and instructions as being on the cover sheet of the current study survey. All the questions were designed to be clear and free of any ambiguity with an aim of increasing response accuracy and reducing CMB. Finally, leveraging Harman’s single- factor test in an attempt to measure CMB, we sought to estimate the proportion of the variance in our underpinning EFA factors, that should always explain the greatest portion of the variance in the indicators, in accordance with two previous works; [4, 57]. A common method bias exists in a study in case of If the total variance extracted by one factor is more than 50%. Evidence of common method bias cannot be reported in this data set for the total variance explained by one factor which is 30.418% and which is within acceptable range of 50%.

### Hypotheses testing

**Fig. 2:**
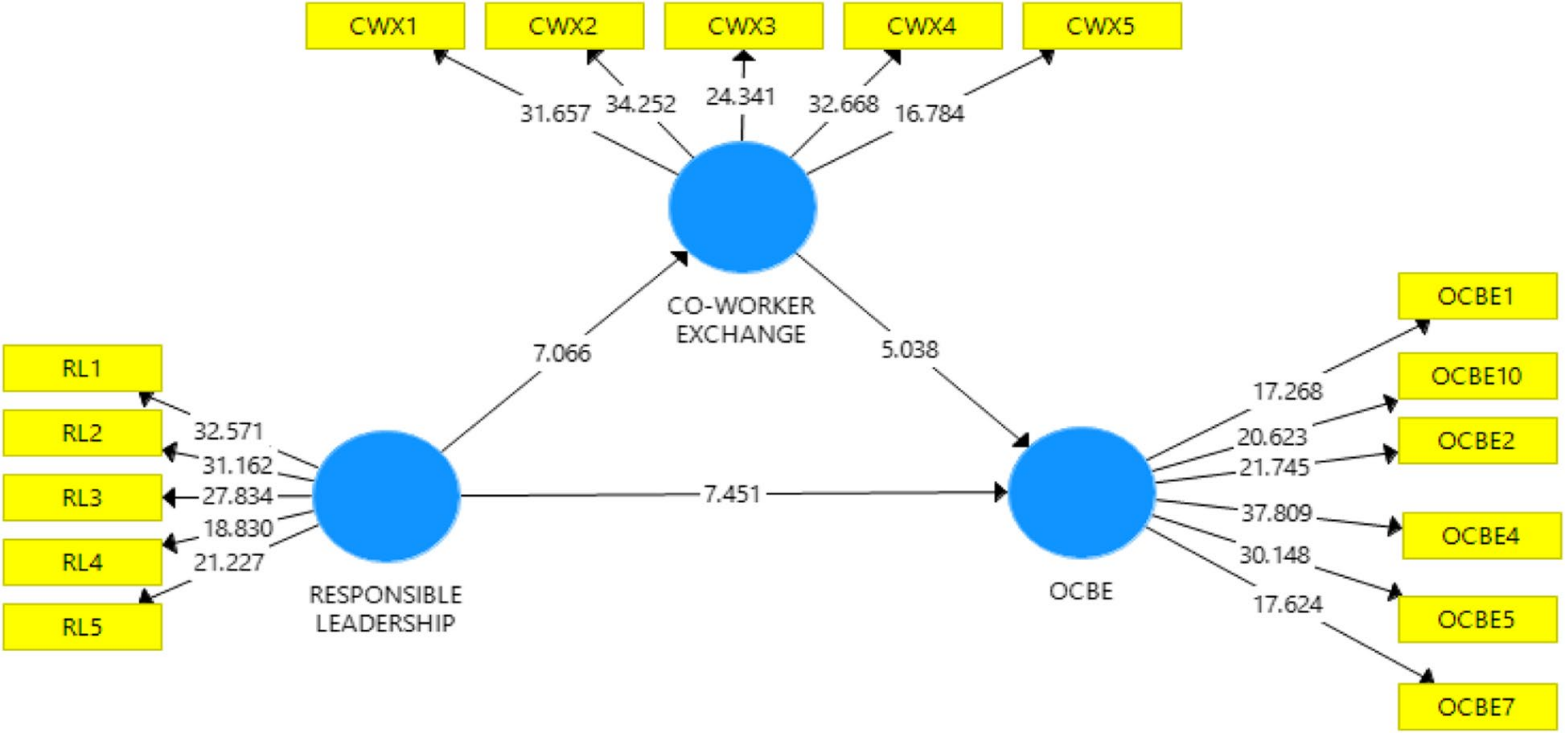
Structural analysis

Testing the current study’s hypothesized relationships using the PLS algorithm in Smart PLS came next. We generated path coefficients, as illustrated in Fig. 2 below. To determine if the path coefficients are statistically significant, this study uses bootstrapping procedures [145] using Smart PLS 3.0. Table 4 illustrates how we used the bootstrapping technique to calculate the T-values for each path coefficient, subsequently producing *P*-values. Hypothesis H1: There is a significant impact of RL on OCBE (β = 0.512, t = 7.539, *p* < 0.001).

**Table 4:** Direct effect results

	Std. Beta	Sample Mean (M)	Standard Deviation (STDEV)	t-Value
Co-worker Exchange -> OCBE	0.357	0.358	0.069	5.038***
RL ->Co-worker Exchange	0.512	0.511	0.068	7.066***
RL -> OCBE	0.46	0.463	0.06	7.451***

***Significant at *p*<0.01

### Testing the mediating effect of co-worker exchange

The study’s theoretical framework hypothesizes that co-worker support mediates the relationship between RL as a construct and OCBE. We used Smart PLS 3.0 to test the mediating effect. Table 5 displays the hypotheses’ outcomes. The results demonstrated that there is a complementary mediation of coworker exchange linking RL and OCBE (β = 0.183, t = 4.623, *p* < 0.001). Therefore, hypothesis H4 was accepted.

**Table 5:** Indirect effect results

	Std. Beta	Sample Mean	Standard Deviation	t-Value	
RL-> Co-Worker Exchange -> OCBE	0.183	0.182	0.04	4.623***	Complimentary Mediation

***Significant at *p*<0.01

### Role of moderating variables

**Fig. 3:**
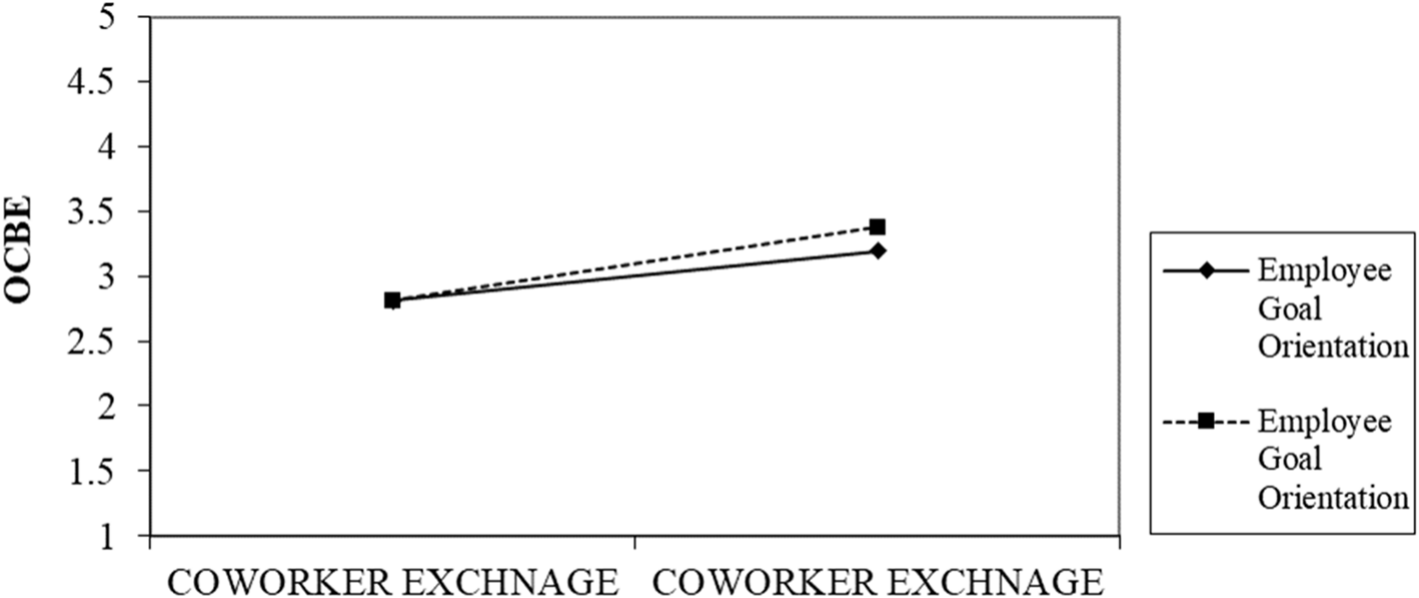
Interaction graph for employee goal orientation as moderator

**Fig. 4:**
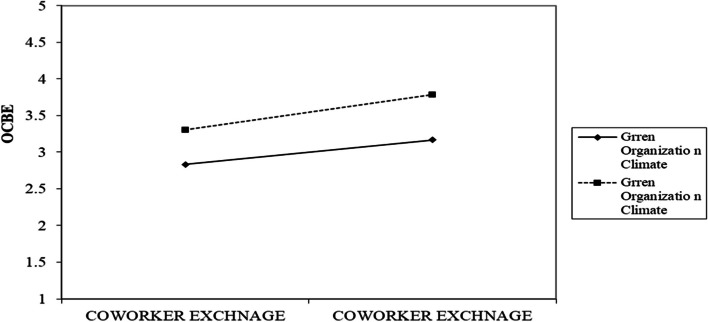
Interaction graph for green organization climate as moderator

**Fig. 5:**
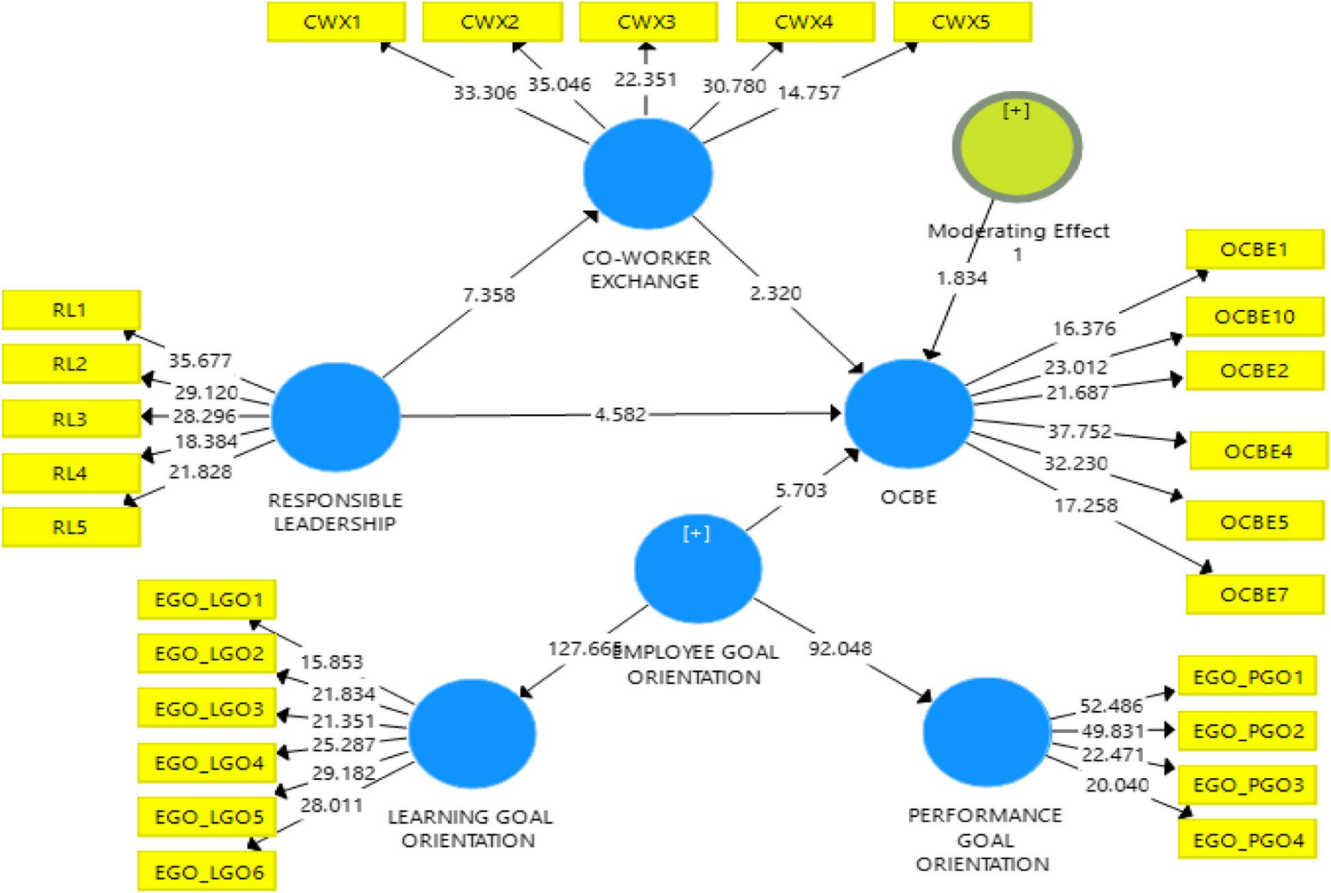
Moderating effect of employee goal orientation

**Fig. 6:**
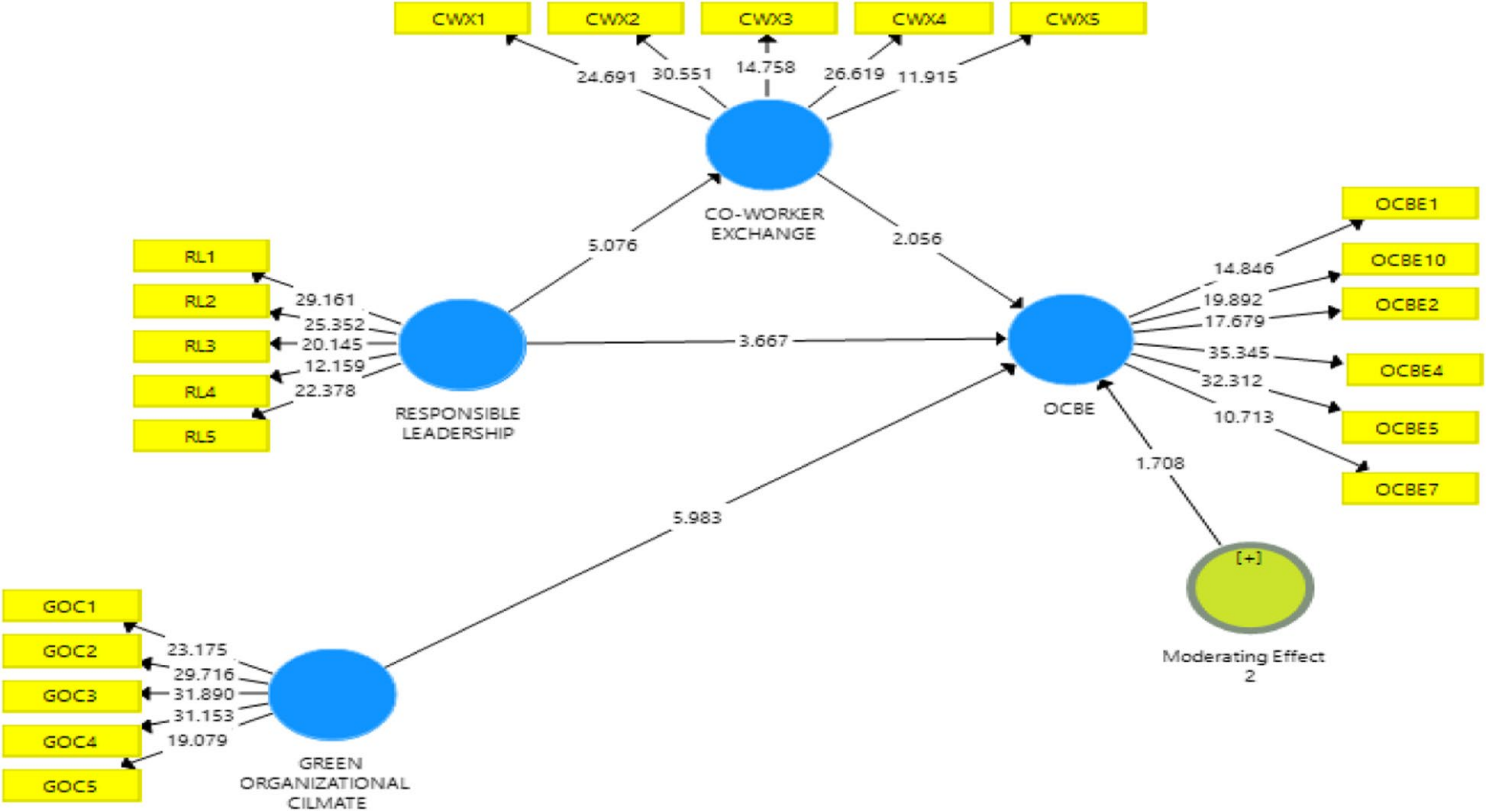
Moderating effect of green organization climate

Researchers proposed three moderating effects between co-worker exchange and OCBE: supervisory support, employee goal orientation, and green organization culture. As shown in Table 6, the results reveal that there exists a moderating impact of employee goal orientation as well as a GROC (β = 0.092, t = 2.013, *p* < 0.01) and (β = 0.074, t = 1.708, *p* < 0.10), respectively. As a result, the study’s hypotheses are supported in H6 and H7, respectively. We also examine the slope gradients to interpret the interaction plots. Employee goal orientation does have a more positive effect when it is high, as seen in Fig. 3, where the line labeled “Employee Goal Orientation” has a steeper gradient than the Low Employee Goal Orientation line. The GROC line in Fig. 4 also exhibits a steeper gradient than the low level of GROC, suggesting that there is a higher positive correlation when GROC is high. Our pre-investigation assumptions support our hypotheses H6 and H7. Figures 5 and 6 display the structural diagrams of the moderating effect for both employee green orientation and GROC. Finally, we found the impact of supervisory support as a moderating variable to be insignificant. Thus, Hypothesis H8 is not supported.

**Table 6:** Moderating effect results

	Std. Beta	Standard Deviation	t-Value	
Employee Goal Orientation -> OCBE	0.092	0.046	2.013**	
Green Organizational Culture -> OCBE	0.074	0.043	1.708*	
Supervisory Support -> OCBE	−0.033	0.066	0.505NS	

*Significant at *p* < 0.10

**significant at *p* < 0.05

*NS* Not Significant

## Discussion & conclusion

### Discussion

This research examines the role of RL as a foundational element of organizational citizenship behavior for the environment (OCBE) and the ways in which it is influenced by coworker exchange relation, drawing from social learning theory and social exchange. The link between RL and OCBE may be influenced by moderators such as a GROC, supervisory support, and employee goal orientation. Despite extensive research on leadership and organizational citizenship behavior (OCB), responsible leadership’s role in fostering organizational citizenship behavior for the environment (OCBE) remains underexplored, particularly in the context of environmental sustainability [2, 167]. This study addresses this gap by investigating how contextual factors, such as coworker interactions and supervisory support, influence the relationship between responsible leadership and OCBE, thus providing a multidimensional perspective that enriches the existing literature. While previous studies have predominantly focused on the direct effects of leadership on organizational outcomes, this research includes coworker exchange as a mediator to explore the social mechanisms that facilitate or hinder the translation of responsible leadership into pro-environmental behaviors [117]. By integrating these dynamics, the study responds to recent calls for more comprehensive models that examine the interplay of multiple factors in influencing sustainability practices within organizations [117]. This integrative approach not only advances theoretical understanding but also offers practical insights into the mechanisms and conditions—such as supervisory support and employee goal orientation—that shape the impact of responsible leadership on OCBE, thereby making a significant contribution to the field.

This study explores why RL impacts employee OCBE through coworker exchange and how various factors influence the relationship between RL and OCBE. The study employed an extended model that included several abstract pieces of evidence, but it lacked empirical exploration [154]. study was one of the first to delve into the relationship between RL and employee OCBE, suggesting the need for further investigation with different mediation and moderation mechanisms. Thus, utilizing key variables from this model, our study aims to investigate the relationship between RL and employee OCBE, with coworker exchange as a mediator and supervisor support, employee goal orientation, and GROC as moderators.

With the exception of the moderation hypothesis, all of our other hypotheses were confirmed by our empirical examinations. Employee OCBE was shown to be favorably connected with RL, according to this research. The positive impact of responsible leadership on organizational citizenship behavior for the environment (OCBE) aligns with prior research, which suggests that leaders who demonstrate ethical and responsible behaviors inspire employees to adopt environmentally friendly practices [55]. Such leaders not only set a moral example but also encourage employees to go beyond formal job requirements to support sustainability efforts [98]. This influence is rooted in the leader’s ability to foster a shared vision of environmental responsibility, motivating employees to contribute actively to environmental goals [137]. Workers are more likely to be environmentally cautious while operating under RL, according to two recent research [167]. They increase their OCBE by trying to mimic their leader’s actions [65]. There is a similarity between RL and OCBE in that both aim to integrate social, economic, and ecological advantages while also considering the interests of stakeholders and combining the ideas of leadership and social responsibility [59]. Working under RL’s supervision increases the likelihood that employees will participate in OCBE and other pro-environmental behaviors. This kind of leadership helps workers feel like they matter and that the company appreciates what they do by taking their interests and those of other stakeholders into account when making decisions. This recognition encourages workers to engage in OCBE and other voluntary behaviors while on the job.

According to the results, RL and a coworker exchange are significantly related [29]. also found a significant association between leadership and coworker exchange. A responsible leader can establish and sustain good relations among all the stakeholders [82]. Workers who get al.ong well with their bosses and see constructive leadership in action are more likely to see similarities amongst themselves and work together more closely. Leaders, official performance appraisers, reward distributors, and mentors of subordinates have the potential to shape the immediate environment of the workgroup and increase the trust of employees in their coworkers to improve their workplace relationships (e.g., CWX) [26].

Cooperation among coworkers mediated the favorable relationship between RL and organizational citizenship behavior toward the organization (OCBE), according to the study’s results. The finding that coworker exchange mediates the relationship between responsible leadership and OCBE is supported by previous studies showing that a supportive coworker environment can enhance the effect of leadership on pro-environmental behaviors [39]. When leaders exhibit responsible behavior, it creates a culture of mutual support and collaboration, leading coworkers to share knowledge and encourage each other to engage in OCBE [34]. This interpersonal dynamic helps translate the ethical values demonstrated by leaders into actual environmental behaviors among employees. If a boss believes in two of their workers, the rest of the workforce will follow suit, according to balance theory [66]. Coworker exchange, according to [69], makes workers feel obligated to help one another, which in turn leads to more altruistic actions. Leadership, according to [85] promotes employee-to-employee communication, which leads to more civic engagement on the job. Positive leadership conduct in the workplace fosters a feeling of belonging among workers, which in turn strengthens connections amongst coworkers. Workers who get al.ong well with their colleagues are more likely to pitch in when needed and go above and beyond to assist the company and its employees.

The results show that goal orientation had a crucial moderating role in the connection between OCBE and communication amongst employees. These findings are consistent with other studies that highlight how individual goals and values influence the extent to which social factors, such as coworker relationships, drive pro-environmental behaviors [154]. According to [92] OCB increases participation from goal-oriented workers since it provides more chances for professional growth. Workers who are goal-oriented (in areas like learning and performance, for example) like to hear good things about their abilities. Participating in OCBE provides them with a wealth of chances to grow and learn. Thus, workers who are more goal-oriented tend to exhibit greater levels of OCBE while on the job. Employees with a strong orientation toward environmental goals are more likely to be positively influenced by coworker support and collaboration, leading to a higher level of engagement in OCBE [93]. This indicates that personal commitment to green objectives strengthens the impact of coworker interactions on environmental actions.

Coworker interchange and organizational citizenship behavior enhancement (OCBE) are both mediated by GROCs, according to this research [169]. found that green workplaces encourage more eco-friendly actions from workers; in other words, a green environment significantly reduces organizational citizenship behavior effect (OCBE). Managers are responsible for informing workers of company policy and providing them with the resources they need to go above and beyond the call of duty [156]. The environment of a company tells workers a lot about its beliefs and goals, as well as the kind of attitudes and activities it wants from them. Employees are motivated to do OCBE more efficiently by a GROC.

Finally, the study set out to discover if and how supervisory support mediated the relationship between OCBE and coworker interaction. Previous research and theories supported this result. If workers believe their bosses have their backs, they’ll go out of their way to assist them out, which boosts their organizational citizenship behavior [21]. This is based on the social exchange theory and the reciprocity norm. This finding aligns with some prior studies that suggest the influence of supervisory support on employee behaviors may not be significant when peer relationships and personal environmental goals are strong drivers [76]. It is possible that, in contexts where coworker exchange is robust, the additional influence of supervisory support is less critical for motivating environmental behaviors, as employees may already feel empowered and supported by their peers [15]. Since our data do not corroborate most of the previous research, a more thorough investigation is required to determine the likely reasons of insignificant correlations. That is to say, employees who value meaningful interactions with their colleagues are more likely to support the initiatives of ethical managers than those who are self-absorbed and think their needs are more important than everyone else’s. When both a goal-oriented environment and a green atmosphere are present, this association grows stronger. These findings deepen our understanding of the context and processes via which RL influences workers’ actions outside of their job descriptions.

### Conclusion

Research like this shows that RL is a must-have for environmental citizenship behavior in the workplace (OCBE), with the positive benefits of RL spreading from one colleague to another. Employees’ goal orientation and the presence of a GROC have a substantial impact on the connection between OCBE and interactions with coworkers. It follows that people’s contacts with colleagues can indirectly affect the positive effect of RL on OCBE. Both theoretical research and real-world businesses may benefit from the insights provided by the results of how RL promotes OCBE.

## Theoretical & managerial implications

### Theoretical implications

Organizational citizenship behavior refers to community involvement that is neither mandated nor included in the formal remuneration system. It is the initiative taken by employees voluntarily to protect the environment. According to [42, 65], companies can only achieve their environmental protection goals via the combined efforts of their employees and their green growth strategies and plans. Research on the effects of RL on OCBE is under underway. We aimed to address the issue, “How does RL influence employee environmental behavior?” by gathering relevant data. as it relates to social learning and the concept of social identity. The idea that subordinates acquire complicated behaviors primarily via seeing and mimicking the actions of those in charge is central to the field of social learning theory [12]. Leadership conduct impacts employee behavior because of the high frequency of interactions between leadership and workers. There is evidence from the past that shows how ethical leadership may improve the ethical climate, which in turn strengthens and improves OCBE. According to [164], their moral exemplarily conduct is also a key factor in this. Leadership styles associated with eco-friendly principles should be the focus of further research, according to [71]. Depending on the circumstances at work, research suggests that leaders have a significant impact on their subordinates’ propensity to learn and mimic their behaviors. By encouraging coworker exchanges that are more focused on environmental preservation, our study suggests that ethical leadership may promote OCBE [74]. In addition, the research looked at how GROC, supervisory support, and employee goal orientation mediated the connection between OCBE and coworker interchange. Additional research is needed to understand how RL affects OCBE, according to [167]. The relationship between RL and OCBE may be moderated by characteristics such as leader support, ambient atmosphere, and employee goal orientation, among others. Our study considerably enhanced the state of the art in the area by diving into these linkages.

### Managerial implications

Motivating workers to give their all is a proven method for increasing a company’s bottom line, according to recent research [65, 92, 142]. To help reduce the increasing environmental impact of the present climate chaos businesses can promote OCBE and other environmentally protective practices [106] among their employees and focus on long-term sustainability. As a result, it is crucial to encourage more eco-conscious actions on the part of employees. Here are some management implications of the current study’s results: Employees’ daily practices in the workplace are affected by the leader’s moral compass and perspective on CSR. By communicating their long-term aims and principles to the present organization, responsible leaders raise the consciousness of their subordinates. In addition, leaders may provide personalized assistance to their subordinates by considering their needs, encouraging personal growth [111], and attentively listening to fresh perspectives. A trusting, responsible, and supportive work environment makes employees feel appreciated and secure enough to engage in OCBE. According to this research, businesses should strive to hire people who are enthusiastic about learning and who are also focused on meeting performance goals. They should also create a culture where executives encourage and support workers to be environmentally conscious. As a result of the positive impression, they get from working in an ecologically conscious atmosphere, workers are more likely to go above and beyond the call of duty to help the company achieve its environmental goals. Managers need specialized training to improve their RL and boost their capacity to improve workplace interactions, which will encourage workers to take action to preserve the environment and, in turn, increase organizational citizenship behavior enhancement (OCBE).

### Research limitations and future directions

There are a few limitations associated with this study to be deliberated. First of all, the instrument used to measure responsible leadership is derivative from scales developed for the western perspective. Scales have good validity and reliability, but the theoretical association of responsible leadership and its endorsement for diverse cultures, particularly the Asian perspective, including, needs further exploration. Second, the current study design is cross sectional, future studies must plan for longitudinal research. Third, future studies must use other mediators such as employee environmental awareness, psychological empowerment and perceived organizational support for sustainability [30] and moderators’ external environmental pressures and employee environmental values. Fourth the data for leadership perspective was evaluated by the employees and not by the leader themselves. In future studies, we call for leaders’ self-evaluation of leadership traits and their impact on employees OCBE.

## Data Availability

Data could be made available at a reasonable request and after approval from the research wing of Firoz Khan Noon Business School, University of Sargodha.
